# Supplementary material for: Modelling low-dimensional interacting brain networks reveals organising principle in human cognition
Source: Netw Neurosci. 2025 May 8;9(2):661–81. doi: 10.1162/netn_a_00434 (PMC12140577; doi:10.1162/netn_a_00434)
Supplement: Supplementary file 1 [file netn-9-2-661-s001.docx]

**Supplementary Material of : “Modelling low-dimensional interacting brain networks reveals organising principle in human cognition”**

We trained the same autoencoder using three different time-by-time spatial patterns of dimension N=62, 500 or 1000 as the inputs (see Methods for more details of AE architecture). For each latent space dimension ranging from 2 to 20, we evaluated the performance of the AE in reconstructing each spatial pattern by measuring the mean squared error (MSE) between the input and the reconstructed pattern. We computed the average MSE across patterns and participants in each fold as well as in each parcellation. In **Figure 2A** we show the mean and standard deviation across 10-folds for each dimension, enabling us to compare the progression of the reconstruction error for each parcellation. We observed that the behaviour was consistent across all three cases, exhibiting a significant decrease in error in the initial dimensions until reaching an elbow point at approximately latent dimension equal to 10, beyond which the error remained relatively constant.


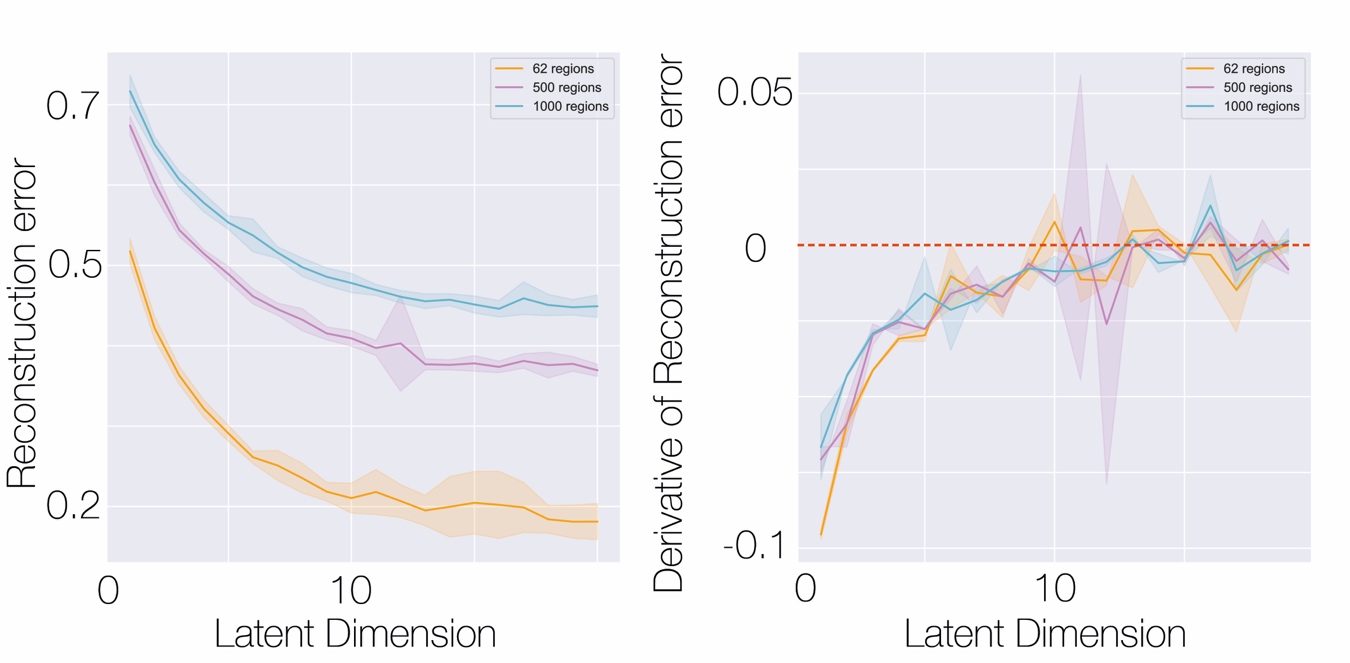

***Supplementary Figure 1. The optimal manifold dimension is invariant respect to the dimensionality of the original state space.***  ***Reconstruction error (left panel).*** *We trained the same AE using as input the time-by-time spatial patterns of 900 fMRI recordings of resting state participants obtained applying three different parcellations the HCP data (DK 62, Schaefer 500 and 1000). We trained and evaluated the AE with a 10-folds scheme randomly selecting the 90%/10% as training and testing sets. We repeated the procedure by changing the latent dimension from 2 to 20 and investigated the reconstruction error, computed as the MSE between each input and reconstructed pattern for 100 participants in the test set. We displayed the mean and standard deviations across folds, and we observed that around latent dimension equal to 10 the MSE presented an elbow for the three parcellations.* ***Derivative of Reconstruction error (right panel)****, we show that the first derivative is close to zero for dimensions around 10 and above demonstrating that the reconstructing error is not changing for high dimensions for all three parcellations.*

## Autoencoders architecture and training

We trained the same autoencoder using three different time-by-time spatial patterns of dimension N=62, 500 or 1000 as the inputs. We created random splits of 90% for training (900 participants) and 10% for testing (100 participants) to obtain separate training and test sets. Then, we trained the AE using a 10-fold cross validation scheme to evaluate the performance of the network. The AE architecture is shown in **Figure 1A**, upper panel. Our encoder network was comprised of a deep neural network that employs rectified linear units as activation functions and consisted of 6 fully connected layers starting with N=1024. This network funnels down to the latent space layer, whose dimensions ranged from 2 to 20. On the other hand, the decoder network mimics the architecture of the encoder, generating reconstructed patters from each encoded point (see Methods for more details of AE architecture). For each latent dimension we evaluated the performance of the AE in reconstructing each spatial pattern by measuring the mean squared error (MSE) between the input and the reconstructed pattern. We computed the average MSE across patterns and participants in each fold as well as in each parcellation.


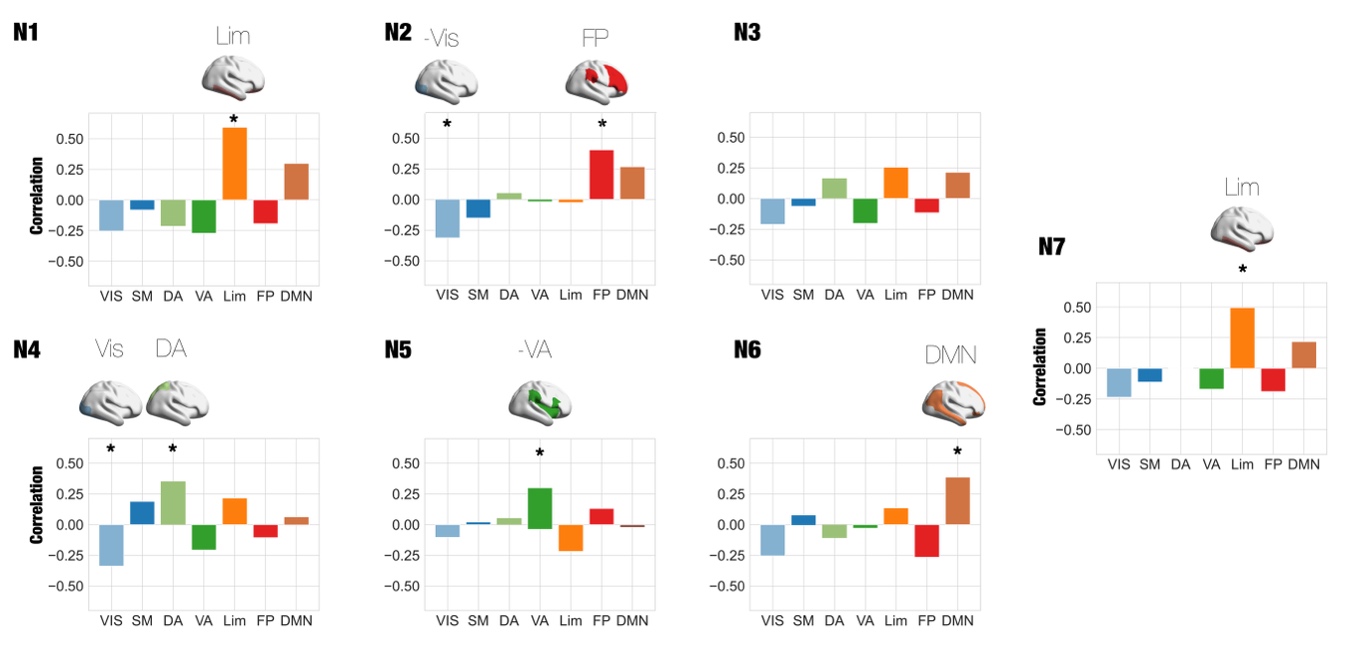


***Supplementary Figure 2. Replication of the results of association between resting state networks and the latent networks revealed by VAE considering seven latent dimensions.*** *We identified each latent mode considering a latens space of dimension 7 with a pattern in the source space of 62 brain regions. We associated each spatial patterns with the Yeo 7 resting state networks by computing the correlation of each pattern with the percentage of belonging of each region to each RSN (* indicates the correlation that are significant after false discovery rate correction). The reference functional brain networks estimated by Yeo and colleagues named: Visual (VIS), Somatomotor (SM), Dorsal Attention (DA), Ventral Attention (VA), Limbic (Lim), Frontoparietal (FP) and Default mode (DMN).*


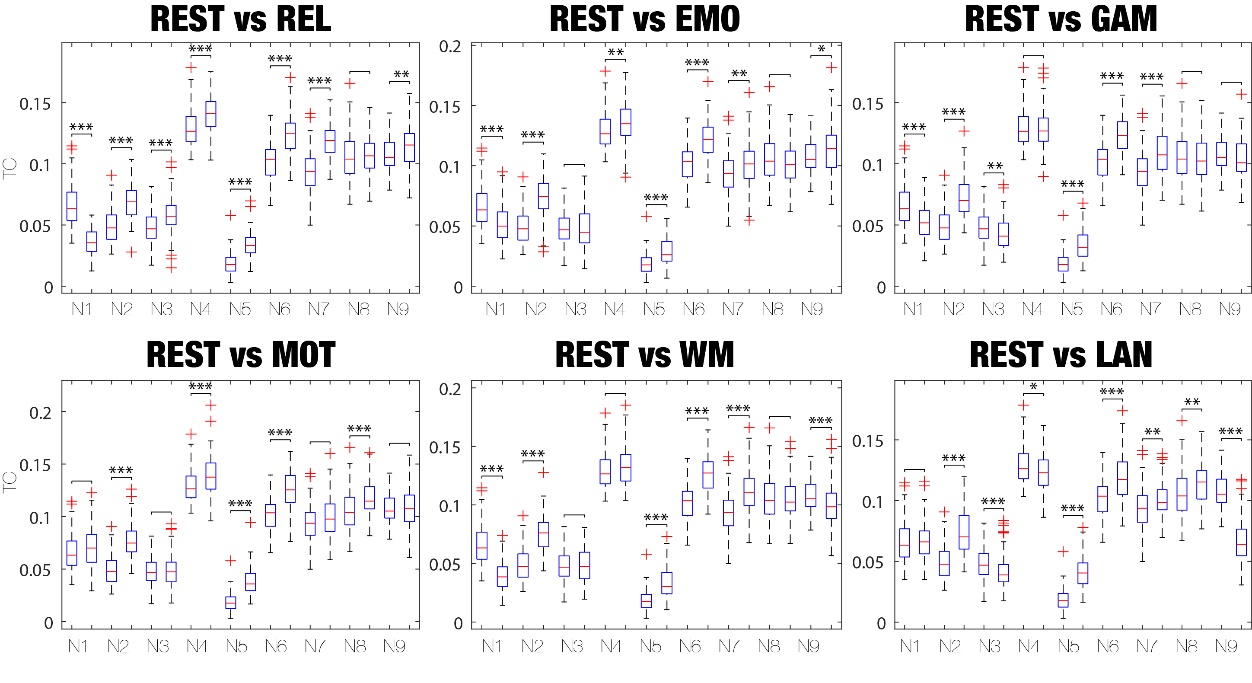


***Supplementary Figure 3. Modelling the low-dimensional manifold network show a flexible reconfiguration of network interaction during cognitive tasks.*** *We computed the total level of connectivity (TC) of each network as the sum of all outcome and income interactions. We found significant differences (Wilcoxon ranksum test, false discovery rate corrected) in the level of TC for almost all networks in the comparison between resting-state and tasks. (*** means p value<0.001;* means 0.01<p value<0.05)*

***Supplementary Table 1. Association between brain regions in DBS 62 and RSN for the left hemisphere.*** *We display a table with the association between brain regions in DBS62 and the 7YEO RSN, for simplicity only for the left hemisphere.*
